# Efficacy of a mobile phone-based life-skills training program for substance use prevention among adolescents: study protocol of a cluster-randomised controlled trial

**DOI:** 10.1186/s12889-018-5969-5

**Published:** 2018-09-10

**Authors:** Severin Haug, Raquel Paz Castro, Andreas Wenger, Michael P. Schaub

**Affiliations:** Swiss Research Institute for Public Health and Addiction, Zurich University, Konradstrasse 32, 8005 Zurich, Switzerland

**Keywords:** Life-skills, Substance use, Prevention, Adolescents, Mobile phone

## Abstract

**Background:**

Life-skills trainings conducted within the school curriculum are effective in preventing the onset and escalation of substance use among adolescents. However, their dissemination is impeded due to their large resource requirements. Life-skills training provided via mobile phones might represent a more economic and scalable approach. The main objective of the planned study is to test the efficacy of a mobile phone-based life-skills training to prevent substance use among adolescents within a controlled trial.

**Methods/design:**

The efficacy of a mobile phone-based life-skills training to prevent substance use among adolescents will be tested in comparison to an assessment only control group, within a cluster-randomised controlled trial with two follow-up assessments after 6 and 18 months. The fully automated program is based on social cognitive theory and addresses self-management skills, social skills, and substance use resistance skills. Participants of the intervention group will receive up to 4 weekly text messages over 6 months in order to stimulate (1) positive outcome expectations, e.g., on using self-management skills to cope with stress, (2) self-efficacy, e.g., to resist social pressure, (3) observational learning, e.g. of interpersonal competences, (4) facilitation, e.g., of strategies to cope with negative emotions, and (5) self-regulation, e.g., by self-monitoring of stress and emotions. Active program engagement will be stimulated by interactive features such as quiz questions, message- and picture-contests, and integration of a friendly competition with prizes in which program users collect credits with each interaction. Study participants will be 1312 students between the ages of 14 and 16 years from approximately 100 secondary school classes. Primary outcome criteria will be problem drinking according to the short form of the Alcohol Use Disorders Identification Test and cigarette smoking within the last 30 days preceding the follow-up assessment at month 18.

**Discussion:**

This is the first study testing the efficacy of a mobile phone-based life-skills training for substance use prevention among adolescents within a controlled trial. Given that this intervention approach proves to be effective, it could be easily implemented in various settings and would reach large numbers of young people in a cost-effective way.

**Trial registration:**

ISRCTN41347061 (registration date: 21/07/2018).

## Background

Several biological, psychological, and social transitions that occur during adolescence are essential for a young person’s later-life trajectory [1, 2]. These transitions offer opportunities for them to gain skills to achieve greater autonomy from adults, build social connections with peers, develop a positive body image, and form a sense of identity. However, these transitions also facilitate exploration and risk taking at a stage when cognitive functions of the brain are not yet fully developed [3]. Shifts of emotional regulations and increased risky behaviours result in vulnerabilities for mental and substance use disorders, which constitute the biggest contributors to the health burden of 10- to 24-year-old individuals [4]. Substance use and the development of substance use disorders often first emerge during adolescence and co-occur with mental disorders [1]. The Swiss data of the Health Behaviour in School-aged Children (HBSC) study [5] showed noticeably increases in the lifetime prevalence of alcohol, tobacco and cannabis consumption over the age groups of 11-to-15-year-olds. The prevalence of regular cigarette smoking (weekly or daily) increased from 2% among 13-year old boys to 12% among 15-year-old boys and from 2% in 13-year old girls to 9% in 15-year old girls. The prevalence of binge drinking increased from 19% in 14-year old boys to 27% in 15-year old boys and from 14% in 14-year old girls to 23% in 15-year old girls.

A systematic review of studies assessing the effectiveness of prevention, early intervention, and harm reduction in young people for tobacco, alcohol, and illicit drugs demonstrated the effectiveness of taxation, public consumption bans, advertising restrictions, and minimum legal age, as well as the potential effectiveness of preventative interventions that deliver life-skills training in educational settings [6]. Schools are particularly suitable settings to reach adolescents with preventative interventions because of the ease of delivery and access to young people within compulsory secondary education. A Cochrane Review on school-based programs for the prevention of tobacco smoking [7] concluded that combined social competence and social influence interventions had a significant effect at 1 year and at longest follow-up (OR 0.49, 95% CI 0.28 to 0.87), whereas a social influences program on its own, multimodal community-wide initiatives, and information-only interventions were found to be ineffective. Another Cochrane review on school-based prevention programs for alcohol misuse in young people [8] concluded that certain generic psychosocial and developmental prevention programs can be effective. However, the methodological quality of the trials included in the analysis was poor, and this did not allow for any quantitative pooling of data.

The majority of the generic programmes addressing social competences and social influences which were included in the above-cited reviews can be described as life-skills trainings and primarily rely on Bandura’s social learning theory [9] which hypothesizes that children and adolescents learn substance use by modelling, imitation, and reinforcement, influenced by individual cognitions, attitudes. Furthermore, based on the social influences approach, [10] substance use susceptibility is increased by poor personal and social skills and young people initiate drug use as a result of pressure from peers, family and the media.

Generic life skills programs to prevent substance use, like the IPSY program, developed in Germany [11] or the ALERT [12] or Life Skills Training [13] programs developed in the US, typically combine training in self-management skills (e.g., coping with stress, emotional self-regulation, goal setting), social skills (e.g., assertiveness, communication skills) and substance use resistance skills (e.g. resisting peer pressure to drink alcohol, recognizing and resisting media influences promoting cigarette smoking, normative expectations about substance use).

Although these life-skills training programs were effective at preventing the onset of specific substances [7, 11, 14] or at decreasing problematic substance use [8], their implementation and dissemination in schools present serious challenges [15]. First, teachers and other professionals need the time, motivation, knowledge and skills to deliver the program. Second, extensive resources, in terms of personnel, money, and time allocated to deliver substance use prevention, are required to prepare and administer such programs.

Electronically-delivered interventions (e.g. via computer, Internet or mobile phone) have the potential to overcome the above-mentioned obstacles that hinder successful program implementation and larger-scale dissemination of life-skills training in schools. Electronically-delivered interventions have a wide reach at a low cost, and offer the opportunity to automatically deliver individually-tailored contents that can be accessed at any time and in any place [16]. Furthermore, electronically-delivered interventions might be more appealing for adolescents, because they can better ensure privacy and tailor contents to their needs.

A recent systematic review of alcohol and other drug prevention programs facilitated by computers or the Internet [17] identified nine trials of online prevention programs, of which six demonstrated significant, but modest effects for alcohol and/or other drug use outcomes. The programs were delivered in the US, Australia and the Netherlands and provided between 1 and 12 online curriculum-based standard lessons or tailored feedback. All programs were universal, i.e., delivered interventions to all students regardless of their level of risk, and were primarily based on principles of the social learning theory [9], the social influences approach [10] and the social cognitive theory [18, 19].

Beyond traditional personal computers, a promising means of delivering prevention programs is to do so remotely through the use of mobile technologies. In Switzerland, as in most other developed countries, almost all (98%) adolescents between the ages of 12 and 19 own a mobile phone, and 97% of these phones are smartphones [20]. Most adolescents are familiar with how to use mobile phones and typically use them on a daily basis for texting, taking pictures, playing games etc. Mobile phone-based interventions can provide almost constant support to users, in comparison to interventions that can only be accessed at specific times or locations and they provide a discrete and confidential means of intervention delivery [21]. Particularly mobile phone text messaging is a suitable means of delivering individually tailored messages via mobile phone. This interactive service allows cost-effective, instantaneous, direct delivery of messages to individuals. Several recent reviews underline the potential and efficacy of text messaging-based interventions in various health domains (e.g., diabetes self-management, weight loss, physical activity, smoking cessation, and medication adherence) and for different target groups, including adolescents and young adults [22–25].

Within a pre-post study in Switzerland, the acceptance and potential effectiveness of a mobile phone-based life-skills training program for substance use prevention among non-smoking vocational school students was tested [26]. This program was based on social cognitive theory and addressed self-management skills, social skills, and substance use resistance skills. Program participants received up to 3 weekly text messages over 6 months. Active program engagement was stimulated by interactive features such as quiz questions, message and picture-contests, and integration of a friendly competition with prizes in which program users collected credits with each interaction. A total of 1067 vocational students who owned a mobile phone and were not regular cigarette smokers were invited to participate in the program. Of these, 877 (82.2%) participated in the program and the associated study. Pre-post comparisons revealed decreased perceived stress and increases in several life skills addressed between baseline and the follow-up assessment. The proportion of adolescents with at-risk alcohol use significantly declined from 20.2% at baseline to 15.5% at follow-up.

Based on these results, showing high-level acceptance and promising effectiveness of a mobile phone-delivered life-skills training program among vocational school students, a reasonable next step is to test the efficacy of this interventional approach within a controlled trial. However, as the age of onset of substance use is strongly increasing between the ages of 14 and 16 [5], it might be even more appropriate to test this universal prevention approach among secondary school students.

Within this study protocol, we describe a cluster randomised controlled trial testing the efficacy of a similar mobile phone-based life-skills training program to prevent substance use among secondary school students.

## Methods/design

### Design and hypotheses

**Fig. 1:**
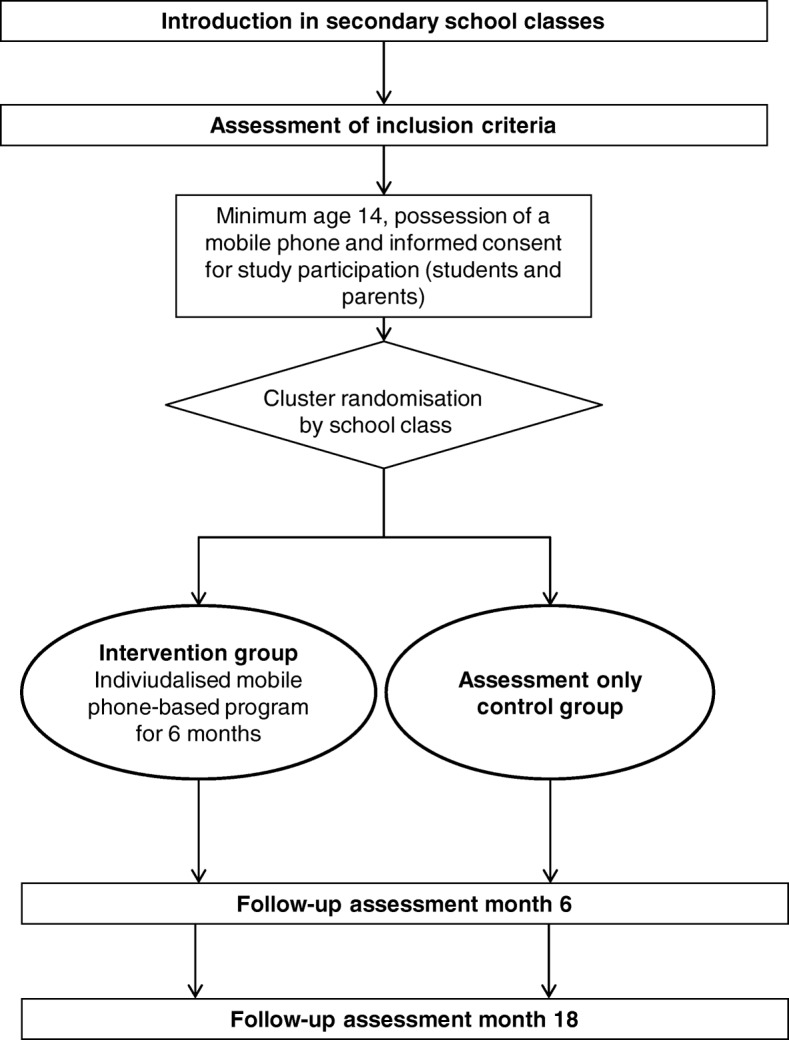
Study design

A two-arm cluster-randomised controlled trial will be conducted to test the efficacy of the *SmartCoach*, a mobile phone-based life-skills training program to prevent substance use among secondary school students. The efficacy of the intervention will be tested in comparison to an assessment only control group. The study participants will be assessed at baseline and at 6- and 18-months follow-up (Fig. 1). Our main hypothesis is that the individually tailored intervention program with a duration of 6 months will be more effective than assessment only, to prevent the onset and escalation of problematic alcohol and tobacco use at 18-months follow-up.

### Participants, setting and procedure

We will test the intervention in secondary school students aged between 14 and 16 (grades 8 and 9). In this age group the prevalence of experimental and regular use of alcohol, tobacco and cannabis is increasing noticeably [5]. However, only a minority has established problematic or disordered substance use [5, 27]. Furthermore, nearly all adolescents at this age are familiar with how to use mobile phones and typically use them on a daily basis. According to the latest representative survey on media use in adolescents in Switzerland, 98% of the adolescents aged 14 and 15 years own a mobile phone and 98% use a mobile phone daily or several times per week [20]. Secondary schools in the German speaking part of Switzerland will be invited to participate in the study by cooperating regional centres for addiction prevention.

Employees of the above-mentioned centres for addiction prevention will arrange information sessions with a duration of 60 min in participating secondary school classes during regular school lessons reserved for health education. These information sessions will be led by junior scientist from the Swiss Research Institute for Public Health and Addiction, who are experienced in the work with young people, the provision of preventive interventions and trained on the study and on the program to be delivered.

The parents of the secondary school students in participating classes will be informed at least 1 week in advance of this session. They will receive a letter including information about the study and the intervention program and the parents are asked to give written informed consent to their child’s participation in the study.

Within the first half of the information sessions in the school classes, the junior scientist will raise awareness on the importance of life skills to effectively cope with the demands and challenges of everyday life. For this purpose, they will use video sequences demonstrating typical stressors and demands for this age group (e.g., search for an apprenticeship, exam stress, peer pressure for substance use) and different strategies to cope with them. The importance of emotional regulation skills and social competences to effectively cope with these stressors will be discussed based on case vignettes. Subsequently, the students will be informed about and invited to participate in a study testing innovative channels for the provision of life-skills trainings. To ensure sufficient participation and, thus, representativeness of the sample [28], a reward of 10 Swiss Francs for participation in each of the two follow-up assessments will be announced.

Students (1) with minimum age 14, (2) who own a mobile phone and (3) providing parental informed consent will be invited to participate in the study. Using their own or a provided mobile phone, informed consent will be obtained online from the study participants. Subsequently, they will be invited to choose a username, to provide their mobile phone number and to fill in the baseline assessment directly on the mobile phone.

Participants of the intervention group will receive additional questions which are necessary for the tailoring of the intervention content. Furthermore, for participants of the intervention group, the mobile phone-based intervention program and its association with a friendly competition will be described in detail. Subsequently, participants of the intervention group will receive an individually tailored web-feedback directly on their mobile phone (see also section intervention). During the subsequent 6 months, participants of the intervention group will receive the individually tailored mobile phone-based life-skills training.

Participants of the assessment only control group will be thanked for their study participation, they will be informed about their group assignment and their reward for participation in the follow-up assessment.

Follow-up assessments after 6 and 18 months will be conducted by a research assistant, within the participating school classes, during regular school lessons and using tablet computers. Computer-assisted telephone interviews will be conducted by a research assistant when assessments cannot take place during a school lesson because of vacations, class resolution, or study participants’ absence from class.

### Ethical review

The study protocol was approved by the Ethics Committee of the Faculty of Arts and Sciences at the University of Zurich (approval number 18.6.5; date of approval June, 21st, 2018). The trial will be executed in compliance with the Helsinki Declaration.

### Randomisation and allocation concealment

To avoid spill-over effects within school classes, we will conduct a cluster-randomised controlled trial using school class as a randomisation unit. Due to the heterogeneity of students in the different secondary schools, we will use a separate randomisation list for each school (stratified randomisation). Furthermore, to approximate equality of sample sizes in the study groups, we will use block randomisation with computer generated randomly permuted blocks of 4 cases [29].

Junior scientists supervising the baseline assessment will be blinded to the group allocation of school classes. In addition, group allocation will not be revealed to participants until they had provided their informed consent, username, mobile phone number, and baseline data. Furthermore, the research assistants who perform the computer-assisted follow up assessments for primary and secondary outcomes will be blinded to the group allocation.

### Sample size calculation

As this is the first study on the efficacy of a mobile phone-based life-skills training for substance use prevention, we could not rely our calculations on conclusive results of similar studies. Instead, our calculations were based on the effect sizes of traditional face-to-face delivered life skills training programs in educational settings. However, to account for the longer duration and higher intensity of traditional life-skills trainings compared to our mobile phone-based training, these estimates based on the effect sizes of traditional face-to-face delivered life skills training programs were revised downwards, resulting in a more conservative estimation.

An estimation of the expectable effect sizes was based on the results of a Cochrane Review on school-based programmes for preventing smoking [7] and on the efficacy of a program for the prevention of binge drinking in adolescents based on the Life Skills Training [30]. The Cochrane Review revealed a statistically significant effect in preventing the onset of smoking for combined social competence and social influences curricula (six RCTs) with an Odds Ratio of 0.49 (95% CI 0.28–0.87). The program for the prevention of binge drinking in adolescence based on the Life Skills Training revealed an intervention effect on problem drinking at the 1-year follow-up, with an odds ratio (OR) of 0.41 (95% CI 0.18–0.93), and at the 2-year follow-up with an OR of 0.40 (95% CI 0.22–0.74). Based on these studies, and a slight downward correction, an OR of 0.60 was expected for the main outcome measures.

Based on an estimated OR of 0.60 and an expected 30-days-prevalence of problem drinking in 16 year-old adolescents of 25% in the control group at the 18-month follow-up (mean of prevalence in 15-year olds derived from HBSC study [5] and in 17-year olds derived from Addiction Monitoring [31]), a sample size of *n* = 370 in each study group would be required to have 80% power for a χ^2^-test (α = 5%, 2-sided) in order to detect this difference based on a calculation using G-Power.

Based on an estimated OR of 0.60 and an expected 30-days prevalence of tobacco smoking in 16 year-old adolescents of 22% in the control group at the 18-month follow-up (mean of prevalence in 15-year olds derived from HBSC study [5] and in 17-year olds derived from Addiction Monitoring [31]), a sample size of *n* = 410 in each study group would be required to have 80% power for a χ^2^-test (α = 5%, 2-sided) in order to detect this difference.

As secondary school students are nested within school classes, we additionally need to consider a potential design effect for the calculation of the sample size for our study. Based on [26, 32], an average cluster size of 13 study participants per school class and an intra-cluster correlation coefficient of 0.05 could be expected. This would result in a design effect of 1.60. Multiplying this design effect by the required size for an unnested sample (*n* = 410) results in a required sample size of *n* = 656 per study group and a total of *n* = 1312 study participants. Thus, based on the participation rates of the previous MobileCoach studies [32, 33] and the pre-post study on a mobile phone-based life- skills training, approximately 100 secondary school classes are required to reach this sample size.

### Intervention program

#### Theoretical background and intervention contents

The intervention elements of the program will be based on the Social Cognitive Theory [18, 19]. This theory relies on the Social Learning Theory, as it was founded on principles of learning within the human social context [9], though it has also integrated several concepts from cognitive psychology. Key concepts of this theory, which will be addressed within the mobile phone-based program are (1) outcome expectations (i.e., beliefs about the likelihood and impact of the consequences of behavioural choices), (2) self-efficacy (i.e., beliefs about one’s personal ability to perform a desired behaviour which could be stimulated, for example, by mastery, experience or persuasion); (3) observational learning (i.e., learning new behaviours via exposure to them through interpersonal or media displays; e.g., through peer modelling); (4) facilitation (i.e., providing strategies, tools, and resources that make new behaviours easier to perform); and (5) self-regulation (i.e., controlling oneself via monitoring, goal-setting, feedback, and self-instruction).

The contents of the mobile phone-based program will rely on proven and widely-disseminated life-skills programs, like IPSY [11], ALERT [12] and Life Skills Training [13]. The program addresses (1) self-management skills, (2) social skills, and (3) substance use resistance skills. The program will be structured according to these major contents with the individually tailored web-based feedback and the mobile phone text messages in weeks 1–9 focusing on self-management skills, the messages in weeks 10–15 focusing on social skills, and the messages in weeks 16–20 focusing on substance use resistance skills. Boosters for each of the components will be provided in weeks 22–24.

#### Technological background

The intervention program will be developed using the MobileCoach system. Technical details of the system are described elsewhere [34, 35]. The MobileCoach system is available as an open source project on http://www.mobile-coach.eu. Password protection and Secure Sockets Layer (SSL) encoding are used to ensure the privacy and safety of data transfer.

#### Individually tailored feedback

The individual tailored web-based feedback will be given to study participants of the intervention group immediately after completion of the online baseline assessment within the school classes. This feedback comprises 4–5 screens, including textual and graphical feedback on stress in general, the individual level of stress in various domains compared to an age- and gender-specific reference group, and individual applied and suggested coping strategies. The feedback will use individual data gathered at the baseline assessment on perceived stress in different domains (school, leisure time, friends, family, and social media) and on individual strategies for coping with stress. Instruments for the assessment of stress and coping strategies will be derived from the study Juvenir 4.0, a national survey on stress in adolescents with more than 1500 participants [36]. Data of this survey will also be used to provide an age- and gender-specific feedback on the individual stress level.

#### Text messages

For a period of 6 months, program participants will receive between two and four individualized text messages per week on their mobile phone. These messages will be generated and sent by the fully-automated system. Within the first 9 weeks, the messages will focus on self-management skills; e.g., coping with stress, emotional self-regulation or management of feelings of anger and frustration. In weeks 10–15, the messages will focus on social skills; e.g., making requests, refusing unreasonable requests, meeting new people. In weeks 16–20, the text messages will focus on substance use resistance skills; e.g., recognizing and resisting media influences, social norms of substance use or the associations of self-management skills and social skills with substance use. Boosters for each of the components will be provided in weeks 22–24. The messages will be tailored according to the individual data from the baseline assessment and on text messaging assessments during program runtime; e.g., on substance use or the individual’s emotional state.

To exploit the full potential of current mobile phones, several interactive features, like quiz questions, tasks to create individually-tailored if-then behaviour plans based on implementation intentions, and message contests, will be implemented within the program. Due to the wide dissemination of smartphones in adolescents [20], several messages will also include hyperlinks to audio files (e.g., audio testimonials, motivational podcasts), as well as to thematically-appropriate video clips, pictures and related websites.

Table 1 shows a selection of the planned intervention elements, which are based on concepts derived from social cognitive theory [18, 19] and the major contents of widely-disseminated life-skills programs [11–13]: self-management skills, social skills, and substance use resistance skills.

**Table 1:** Exemplary intervention elements of the mobile phone-based life-skills training

Concept derived from social cognitive theory	Content category		
	Self-management skills	Social skills	Substance use resistance skills
Outcome expectations	Individually tailored information on pros of using self-management skills to cope with stress.	Tailored information on the pros of applying social skills, e.g., making requests.	Information on pros of resisting alcohol, including social norms within an age- and gender-specific reference group.
Self-efficacy	Videos demonstrating easily applicable strategies to cope with negative emotions.	Contest on creating a bubble text on refusing an unreasonable request within a given situation presented on a picture.	Creation of if-then-plan for resisting invitation for smoking marijuana
Observational learning	Interactive message contest on stress regulation strategies.	Video showing exemplary behaviour on individually poorly developed social skills.	Video podcast showing peer models who successfully refused cigarette smoking.
Facilitation	Creation of if-then-plans for coping with the currently most severely affecting stress situation.	Podcasts introducing conversational skills or strategies for making requests.	Contest on creating a bubble text on refusing an alcoholic drink at a party presented on a given picture.
Self-regulation	Monitoring of and feedback on individual emotions.	Monitoring of and feedback on progress of individual social skills.	Monitoring of and feedback on individual alcohol use.

#### Prize draw

To stimulate active program engagement, program use will be associated with a friendly competition, which will allow program users to collect credits for each interaction (e.g., answering monitoring text messages, participating in quizzes, creating messages or pictures within contests, accessing video links integrated in text messages). The more credits participants will collect, the higher their chances will be of winning one of several attractive prizes which will be part of a prize draw (10 prizes with the sum of 500 Swiss Francs) after program completion. Participants will be able to retrieve their number of credits compared to the number of credits of other program participants’ of their group (similar starting date) at any time from an individual profile page.

### Assessments and outcomes

At baseline, demographic variables (age, sex, migration background), data on mobile phone use as well as characteristics of the schools (location, type of school) and school classes (level, size, number of students present) will be assessed.

Baseline- and follow-up assessments will include

1. Problem drinking in the preceding 30 days, assessed by the short form of the Alcohol Use Disorders Identification Test, the AUDIT-C [37]. This test comprises 3 items on (1) frequency of alcohol consumption, (2) quantity of alcohol consumption, and (3) binge drinking. Pictures will be used to illustrate the quantity of a standard drink, which corresponded to 12–14 g of pure alcohol. Based on a validation study of a large German sample, a cut-off of ≥5 will be used [38].
2. 30-days point prevalence rate for smoking abstinence (“not having smoked a puff” within the past 30 days according to the criteria of the Society for Nicotine and Tobacco Research [39]).
3. Quantity of cigarettes smoked in the preceding 30 days by assessing the number of smoking days and the typical number of cigarettes smoked per smoking day.
4. Cannabis use in the preceding 30 days assessed by an item of the HBSC study [5] addressing the number of cannabis consumption days.
5. Perceived stress assessed by a four-item version [40] of the Perceived Stress Scale [41]. This scale measures the degree to which students appraised situations as stressful over the preceding month.
6. Interpersonal competences, assessed by the brief version of the Interpersonal Competence Questionnaire [42], addressing the following domains of social competence: (1) initiation of relationships, (2) negative assertion, (3) disclosure of personal information, (4) emotional support, and (5) conflict management.

The primary outcomes of the planned study are (1) prevalence of problem drinking in the preceding 30 days according to the AUDIT-C and (2) prevalence of cigarette smoking in the preceding 30 days (having smoked at least a puff according to the criteria of the Society for Nictotine and Tobacco Research [39]).

Secondary outcomes are

- Prevalence of cannabis use in the preceding 30 days (having used cannabis at least once)
- Quantity of alcohol use in the preceding 30 days
- Quantity of cigarettes smoked in the previous 30 days
- Frequency of cannabis use in the preceding 30 days
- Perceived stress
- Interpersonal competences

### Data analyses

Generalized Linear Mixed Models will be used to test intervention effects for binary outcomes and linear mixed models for continuous outcomes [43, 44]. These models account for both fixed and random effects and are particularly useful in analysing longitudinal and nested data (e.g., time within students, students within school classes). To test the efficacy of the intervention, we will test the variables “study group”, “time” and their interaction “study group x time” as predictors of the outcome criteria assessed at follow-up. If necessary, we will control for baseline differences by adding additional baseline variables as covariates to the models. We will conduct both (1) intention to treat analyses and (2) complete case analyses considering all study participants with available follow-up data. For ITT analyses, we will use multiple imputation procedures as described elsewhere [45].

## Discussion

Substance use in adolescents and later adulthood and its related consequences represent a serious public health problem [27]. Although life-skills trainings were effective in preventing the onset and escalation of both problematic alcohol and tobacco use [7, 11, 30], the implementation and dissemination of the existing programs in schools represent serious challenges as they require large resources in terms of money and time [15]. In contrast to comprehensive school-based curricula, training and counselling via mobile phone is more economic and matches with the lifestyle and communication habits of the target group. Most adolescents are familiar with how to use mobile phones and typically use them on a daily basis for texting, taking photos, playing games etc. This is the first study testing the efficacy of a mobile phone-delivered life-skills training for substance use prevention among adolescents within a controlled trial.

Given that this program proves to be effective, it could be disseminated to various groups of adolescents, e.g. in schools or leisure time settings. A translation of the intervention content into other languages would easily enable program dissemination to adolescents in other regions and countries.

## Abbreviation

AUDIT-C: Short form of the alcohol use disorders identification test
